# Repurposing calcium-sensing receptor agonist cinacalcet for treatment of CFTR-mediated secretory diarrheas

**DOI:** 10.1172/jci.insight.146823

**Published:** 2021-02-22

**Authors:** Apurva A. Oak, Parth D. Chhetri, Amber A. Rivera, Alan S. Verkman, Onur Cil

**Affiliations:** 1 Department of Pediatrics and; 2 Departments of Medicine and Physiology, University of California, San Francisco, California, USA.

**Keywords:** Gastroenterology, Therapeutics, Drug therapy, Epithelial transport of ions and water

## Abstract

Diarrhea is a major cause of global mortality, and outbreaks of secretory diarrhea such as cholera remain an important problem in the developing world. Current treatment of secretory diarrhea primarily involves supportive measures, such as fluid replacement. The calcium-sensing receptor (CaSR) regulates multiple biological activities in response to changes in extracellular Ca^2+^. The FDA-approved drug cinacalcet is an allosteric activator of CaSR used for treatment of hyperparathyroidism. Here, we found by short-circuit current measurements in human colonic T84 cells that CaSR activation by cinacalcet reduced forskolin-induced Cl^–^ secretion by greater than 80%. Cinacalcet also reduced Cl^–^ secretion induced by cholera toxin, heat-stable *E*. *coli* enterotoxin, and vasoactive intestinal peptide (VIP). The cinacalcet effect primarily involved indirect inhibition of cystic fibrosis transmembrane conductance regulator–mediated (CFTR-mediated) Cl^–^ secretion following activation of CaSR and downstream phospholipase C and phosphodiesterases. In mice, cinacalcet reduced fluid accumulation by more than 60% in intestinal closed loop models of cholera and traveler’s diarrhea. The cinacalcet effect involved both inhibition of CFTR-mediated secretion and stimulation of sodium-hydrogen exchanger 3–mediated absorption. These findings support the therapeutic utility of the safe and commonly used drug cinacalcet in CFTR-dependent secretory diarrheas, including cholera, traveler’s diarrhea, and VIPoma.

## Graphical abstract

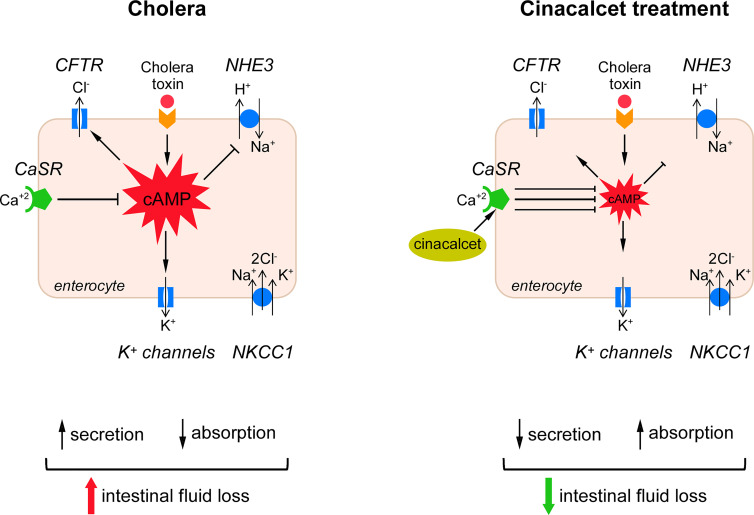

## Introduction

Diarrhea is a major cause of morbidity worldwide and of mortality in the developing world. Globally, diarrhea was responsible for 1.6 million deaths in 2016, with 90% of diarrheal deaths occurring in sub-Saharan Africa and south Asia, and in these regions more than 25% of deaths in children under 5 years of age were due to diarrheal illness (1). Secretory diarrhea is a common type of diarrhea with diverse etiologies including certain bacterial and viral infections, intestinal inflammation, drugs, tumors, and genetic disorders (2). In secretory diarrhea activation of Cl^–^ channels at the luminal membrane of intestinal epithelial cells (enterocytes), including cystic fibrosis transmembrane conductance regulator (CFTR) and Ca^2+^-activated Cl^–^ channels (CaCCs), increases fluid secretion (2). In addition, inhibition of apical membrane sodium-hydrogen exchanger 3 (NHE3) by elevated cyclic nucleotides (3) may contribute to intestinal fluid losses by impairing fluid absorption, as might inhibition of apical anion exchangers (such as SLC26A3) and/or epithelial sodium channel (ENaC) (2).

Cholera and traveler’s diarrhea are major secretory diarrheas caused by the bacterial enterotoxins cholera toxin and heat-stable *E*. *coli* enterotoxin, respectively, whose mechanism involves activation of enterocyte CFTR by phosphorylation following elevation of intracellular cyclic nucleotides (4). Current treatment for secretory diarrhea is primarily supportive, with fluid replacement using oral rehydration solution (ORS) as the mainstay of cholera treatment. Despite ORS, cholera was responsible for more than 100,000 deaths in 2016 (1), and cholera outbreaks can sometimes last for many years due to lack of sanitation infrastructure and access to safe water, which may be logistically challenging in war and disaster zones (5). There is an unmet need for simple, safe, and effective drug treatments for secretory diarrheas.

A proposed target for treatment of secretory diarrheas is the extracellular calcium-sensing receptor (CaSR). CaSR is a G protein–coupled receptor expressed in parathyroid gland, kidney, thyroidal C cells, brain, and bone, as well as throughout the gastrointestinal tract in epithelial cells and enteric neurons (6). Several lines of evidence suggest that CaSR activation might inhibit intestinal fluid secretion and promote fluid absorption, with a variety of mechanisms having been proposed to account for these actions in perfused rat colonic crypts, including activation of phospholipase C (PLC) and phosphodiesterases (PDEs) (7, 8).

The CaSR can be activated by organic cations (Ca^2+^, Mg^2+^, Gd^3+^, etc.), certain heavy metals, amino acids, polyamines, and small molecules, the latter including calcimimetics (CaSR activators) (9). Targeting CaSR in the parathyroid gland by the FDA-approved drug cinacalcet or other calcimimetics suppresses parathyroid hormone (PTH) secretion, which is commonly used to treat secondary hyperparathyroidism associated with chronic kidney disease or renal failure. Cinacalcet is also used to treat hyperparathyroidism and hypercalcemia associated with parathyroid gland tumors (10). Here, we provide evidence that activation of CaSR by cinacalcet in a human colonic cell line and in mouse intestine inhibits intestinal fluid secretion and enhances fluid absorption, offering a simple, novel, and rapidly translatable therapeutic strategy for secretory diarrheas, including cholera.

## Results

### CaSR activation by cinacalcet inhibits CFTR-mediated Cl^–^ secretion in T84 cells.

**Figure 1:**
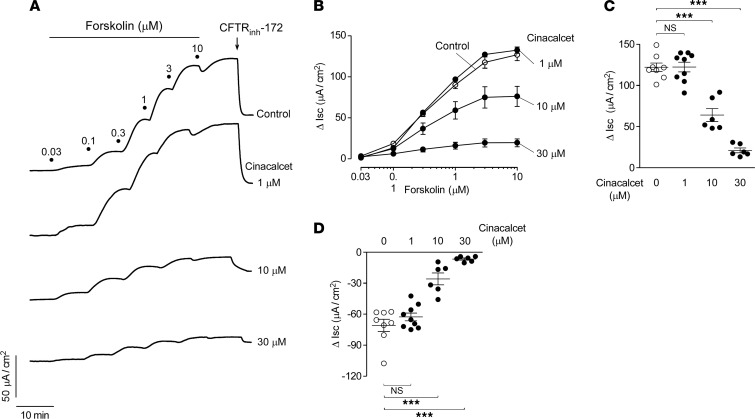
Cinacalcet inhibits forskolin-induced Cl^–^ secretion in T84 cells. (**A**) Short-circuit current (I_SC_) traces showing forskolin concentration response and CFTR_inh_-172 (10 μM) inhibition following 20-minute pretreatment with indicated concentrations of cinacalcet. (**B**) Summary of changes in I_SC_ (Δ I_SC_) from experiments as in **A**. (**C**) Δ I_SC_ induced by maximal (10 μM) forskolin at different cinacalcet concentrations. (**D**) Δ I_SC_ following CFTR inhibitor CFTR_inh_-172 (10 μM) treatment at different cinacalcet concentrations. *n* = 6–9 experiments per group. Mean ± SEM, 1-way ANOVA with Newman-Keuls multiple comparisons test, ***P < 0.001.

Cl^–^ secretion was measured by short-circuit current (I_SC_) in T84 cells, a commonly used human intestinal epithelial cell line that expresses CaSR (11). Though cinacalcet did not alter baseline I_SC_, it largely inhibited the increase in I_SC_ following cAMP elevation by forskolin, which activates CFTR to produce a Cl^–^ secretory current (Figure 1, A and B). The forskolin-induced increase in I_SC_ was inhibited by approximately 50% and 85% by pretreatment of the T84 cells with 10 μM and 30 μM cinacalcet, respectively (Figure 1C), which is consistent with its in vitro EC_50_ of 2.8 μM (12).

The forskolin-induced increase in I_SC_ was partially reversed by the CFTR-selective inhibitor CFTR_inh_-172 (Figure 1, A and D). Cinacalcet pretreatment strongly inhibited the CFTR_inh_-172–sensitive I_SC_ in a concentration-dependent manner. These findings support CFTR inhibition as a major mechanism of cinacalcet action. Similarly, pretreatment of T84 cells with the experimental CaSR agonist R-568 reduced the forskolin-induced maximal I_SC_ increase (Supplemental Figure 1, A and B; supplemental material available online with this article; https://doi.org/10.1172/jci.insight.146823DS1). R-568 appeared to be more potent than cinacalcet in T84 cells with approximately 70% reduction in I_SC_ at 10 μM, which is consistent with its greater reported in vitro potency for CaSR activation compared with cinacalcet (12). As found with cinacalcet, R-568 pretreatment inhibited the CFTR_inh_-172–sensitive I_SC_ (Supplemental Figure 1, A and C). Although R-568 inhibited CFTR-mediated Cl^–^ secretion in T84 cells with modestly greater in vitro potency, subsequent studies were done with cinacalcet because it is an FDA-approved drug with favorable safety profile.

### Cinacalcet-induced CFTR inhibition is indirect and dependent on CaSR.

**Figure 2:**
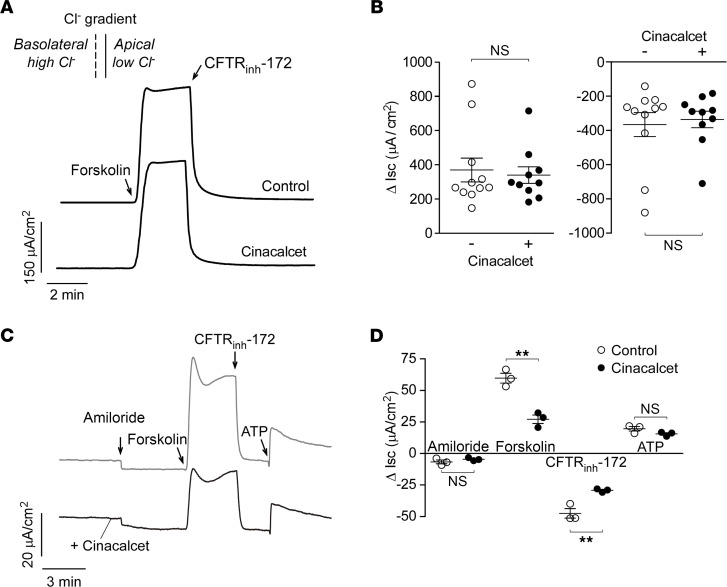
Cinacalcet inhibition of CFTR-mediated Cl^–^ secretion is indirect and depends on CaSR. (**A**) I_SC_ traces in Fischer rat thyroid cells expressing CFTR, showing responses to forskolin (10 μM) and CFTR_inh_-172 (10 μM) following 20-minute pretreatment without and with 30 μM cinacalcet. The basolateral cell membrane was permeabilized with amphotericin B (250 μg/mL) for 30 minutes, and measurement was done using a 60 mM basolateral-to-apical Cl^–^ gradient. (**B**) Summary of data from experiments as in **A** showing Δ I_SC_ with forskolin followed by CFTR_inh_-172. *n* = 10–11 experiments per group. (**C**) I_SC_ in human bronchial epithelial (HBE) cells sequentially treated with amiloride (20 μM), forskolin (20 μM), CFTR_inh_-172 (10 μM), and ATP (100 μM) without and with 20-minute pretreatment with 30 μM cinacalcet. (**D**) Summary of Δ I_SC_ from experiments as in **C**. *n* = 3 experiments per group. Mean ± SEM, Student’s *t* test, **P < 0.01.

To test whether the cinacalcet effect in T84 cells may be due to direct CFTR inhibition, I_SC_ measurements were done in CFTR-transfected Fischer rat thyroid (FRT) cells, which do not express CaSR (13, 14). These cells show a robust cAMP-dependent Cl^–^ current and have been widely used to identify and characterize CFTR modulators (15). In permeabilized FRT cells with a basolateral-to-apical Cl^–^ gradient, cinacalcet pretreatment did not affect CFTR-mediated Cl^–^ conductance as evidenced by the unchanged I_SC_ responses to maximal forskolin and CFTR_inh_-172 (Figure 2, A and B).

The cinacalcet effect was also studied in well-differentiated human bronchial epithelial (HBE) cells, which like T84 cells express CaSR (16), as well as the ion channels CFTR, ENaC, and CaCC (17). Cinacalcet pretreatment had no significant effect on ENaC or CaCC activities, as evidenced by comparable I_SC_ responses to amiloride and ATP, respectively (Figure 2, C and D). However, as found in T84 cells, CFTR-mediated Cl^–^ secretion was largely inhibited by cinacalcet, as seen by the reduced responses to forskolin and CFTR_inh_-172.

**Figure 3:**
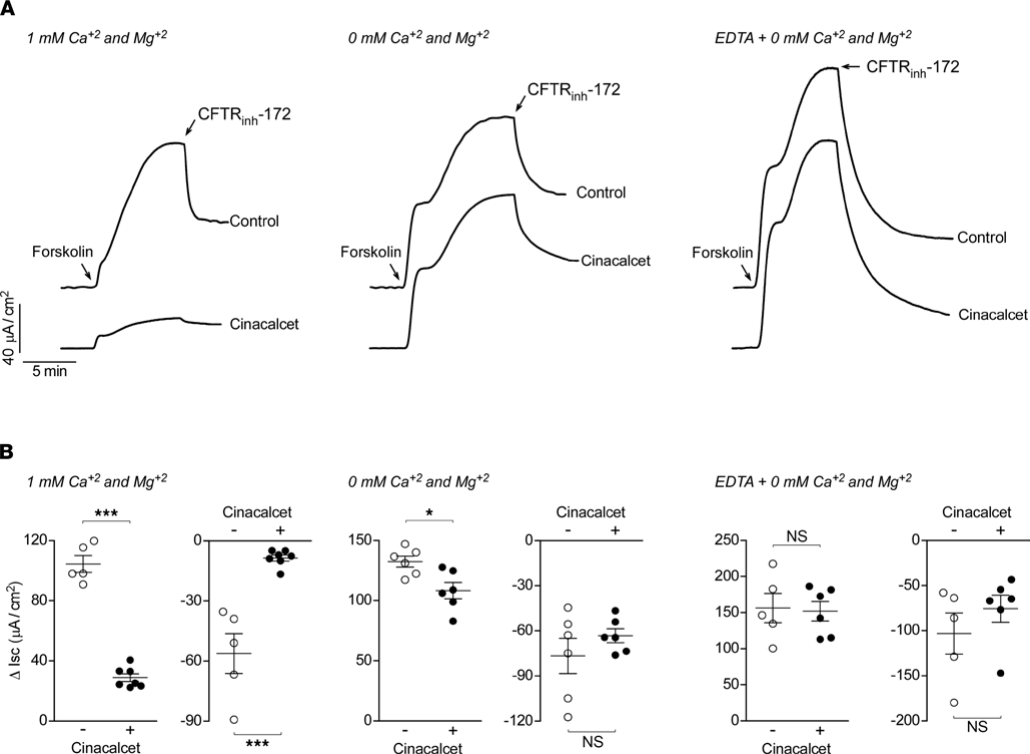
Inhibitory effect of cinacalcet on CFTR-mediated Cl^–^ secretion in T84 cells is dependent on extracellular Ca^2+^. (**A**) I_SC_ traces showing responses to maximal forskolin (10 μM) and CFTR_inh_-172 (10 μM) following pretreatment without and with 30 μM cinacalcet in bathing solutions containing 1 mM Ca^2+^ and Mg^2+^ (left), 0 mM Ca^2+^ and Mg^2+^ (middle), or 0 mM Ca^2+^ and Mg^2+^ plus 1 mM EDTA (Ca^2+^ and Mg^2+^ chelator, right). (**B**) Summary of data from experiments as in **A** showing Δ I_SC_ with forskolin followed by CFTR_inh_-172 for each condition. *n* = 5–7 experiments per group. Mean ± SEM, Student’s *t* test, *P < 0.05, ***P < 0.001.

Type II calcimimetics such as cinacalcet are allosteric modulators of CaSR that increase its sensitivity to extracellular Ca^2+^ and so do not activate CaSR in the absence of extracellular Ca^2+^ (9, 12). To further support the conclusion that the cinacalcet effect in T84 cells is dependent on CaSR activation, forskolin-induced Cl^–^ secretion was measured using Ca^2+^- and Mg^2+^-free bathing solution. In this setting, 30 μM cinacalcet had only minimal effect on the forskolin-induced secretory response and its reversal by CFTR_inh_-172 (Figure 3, A and B), compared with the large responses in cells in the presence of 1 mM Ca^2+^ and Mg^2+^. Since Ca^2+^ and Mg^2+^ are stored intracellularly, we hypothesized that leakage of intracellular Ca^2+^ or Mg^2+^ into the bathing solution might be responsible for the small residual effect of cinacalcet on the forskolin response in the Ca^2+^- and Mg^2+^-free solution. In T84 cells, cinacalcet’s effect on forskolin and CFTR_inh_-172 responses was completely abolished in Ca^2+^- and Mg^2+^-free solution with 1 mM EDTA, a chelator of Ca^2+^ and Mg^2+^. These results in FRT, HBE, and T84 cells indicate that CFTR inhibition by cinacalcet is indirect and dependent on CaSR activation.

### Cinacalcet exerts its antisecretory effect from the apical side in T84 cells.

**Figure 4:**
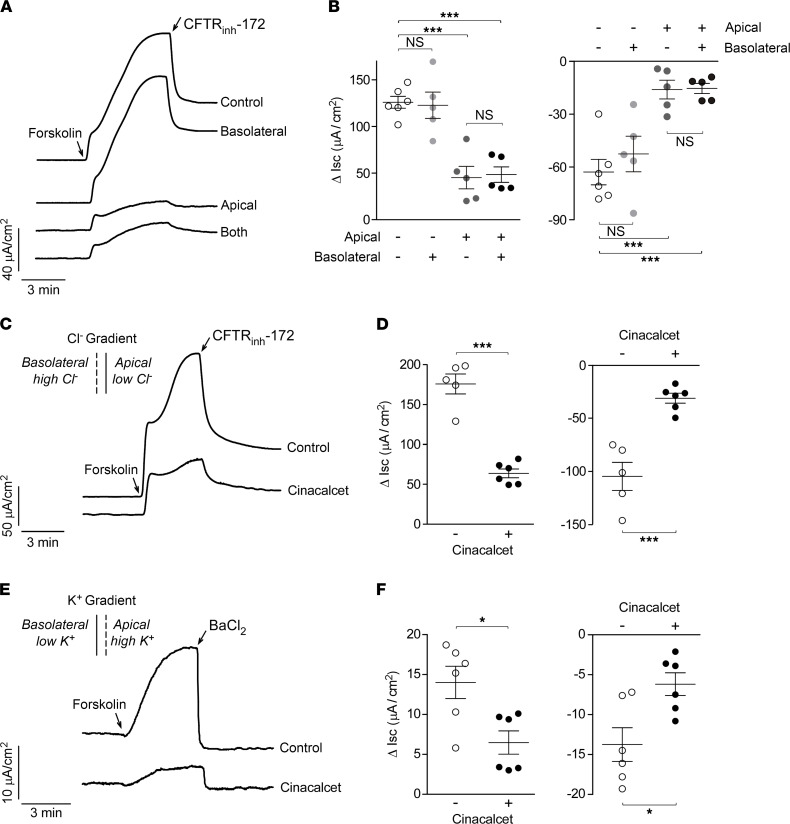
Cinacalcet acts from the apical side in T84 cells for inhibition of apical membrane CFTR-mediated Cl^–^ secretion and inhibits cAMP-activated basolateral membrane K^+^ conductance. (**A**) I_SC_ traces showing effects of 10 μM forskolin and 10 μM CFTR_inh_-172 with 20-minute pretreatment with 30 μM cinacalcet applied from apical, basolateral, or both sides. (**B**) Summary of data from experiments as in **A** showing Δ I_SC_ following forskolin and CFTR_inh_-172. (**C**) I_SC_ traces with basolateral permeabilization (amphotericin B, 250 μg/mL) and 60 mM basolateral-to-apical Cl^–^ gradient showing responses to 10 μM forskolin and 10 μM CFTR_inh_-172 without and with 20-minute pretreatment with 30 μM cinacalcet. (**D**) Summary of experiments as in **C** showing Δ I_SC_ following forskolin and CFTR_inh_-172. (**E**) I_SC_ traces with apical permeabilization (amphotericin B, 20 μΜ) and apical-to-basolateral K^+^ gradient showing responses to 10 μM forskolin and 5 mM barium chloride (BaCl_2_, cAMP-activated K^+^ channel inhibitor, added to basolateral side) without and with 20-minute pretreatment with 30 μM cinacalcet. (**F**) Summary of experiments as in **E** showing Δ I_SC_ following forskolin and BaCl_2_. *n* = 5–6 experiments per group. Mean ± SEM, 1-way ANOVA with Newman-Keuls multiple comparisons test (**B**), and Student’s *t* test (**D** and **F**), *P < 0.05, ***P < 0.001.

CaSR expression has been reported in both apical and basolateral membranes of intestinal epithelial cells (18). In T84 cells, addition of cinacalcet to the apical chamber inhibited the forskolin-induced increase in I_SC_ to a comparable extent to its addition to both the apical and basolateral chambers (Figure 4, A and B). No inhibition of the forskolin-induced I_SC_ was seen when cinacalcet was added to the basolateral chamber only. Similarly, cinacalcet pretreatment reduced the CFTR_inh_-172 effect when added to the apical or both chambers, but not to the basolateral chamber alone.

### Cinacalcet inhibits apical membrane Cl^–^ and basolateral membrane K^+^ conductance in T84 cells.

Forskolin-induced Cl^–^ secretion in intestinal cells involves the coordinated action of several transporters, including apical membrane CFTR, and basolateral membrane K^+^ channels and cotransporters (4). To ascribe the cinacalcet inhibition of I_SC_ in T84 cells to an action on CFTR, I_SC_ was measured in T84 cells following selective permeabilization of the basolateral membrane in the presence of a basolateral-to-apical Cl^–^ gradient, in which I_SC_ provides a direct measure of apical membrane Cl^–^ conductance (19). In this setting cinacalcet pretreatment largely inhibited forskolin-induced Cl^–^ secretion and the CFTR_inh_-172 effect (Figure 4, C and D).

To investigate possible cinacalcet effect on basolateral membrane K^+^ channels, I_SC_ was measured in T84 cells following selective permeabilization of the apical membrane in the presence of an apical-to-basolateral K^+^ gradient, in which I_SC_ provides a direct measure of basolateral membrane K^+^ conductance (20). In this setting, forskolin induced a K^+^ secretory current that was fully inhibited by the BaCl_2_ (cAMP-activated K^+^ channel inhibitor, Figure 4, E and F). Cinacalcet pretreatment substantially reduced basolateral membrane K^+^ conductance in T84 cells as seen by the reduced forskolin- and BaCl_2_-induced I_SC_ changes (Figure 4, E and F). These results support a dual cinacalcet effect in T84 cells involving inhibition of apical membrane CFTR Cl^–^ channel as well as basolateral membrane K^+^ channels.

### Cinacalcet inhibits Cl^–^ secretion in T84 cells induced by cholera toxin, heat-stable *E. coli* enterotoxin, and vasoactive intestinal peptide.

**Figure 5:**
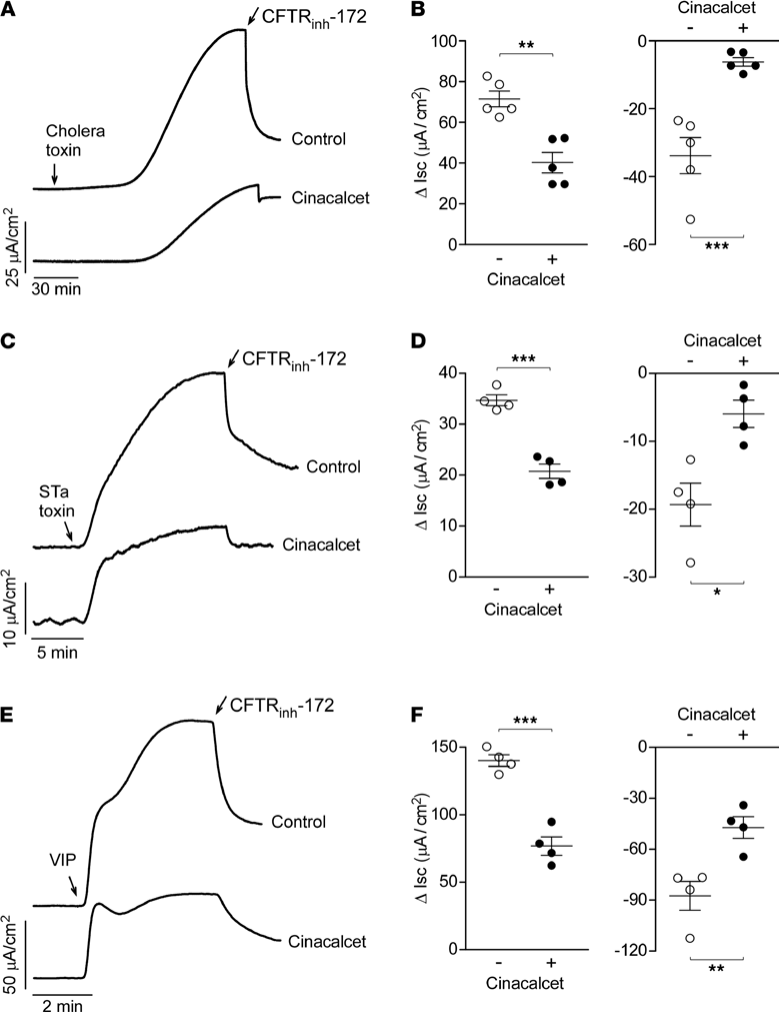
Cinacalcet inhibits cholera toxin–, STa toxin–, and vasoactive intestinal peptide–induced Cl^–^ secretion in T84 cells. (**A**) I_SC_ traces showing responses to 1 μg/mL cholera toxin and 10 μM CFTR_inh_-172 without and with 30 μM cinacalcet pretreatment for 20 minutes. (**B**) Summary of experiments as in **A** showing Δ I_SC_ following cholera toxin (left) and CFTR_inh_-172 (right). (**C**) I_SC_ traces showing responses to 0.1 μg/mL STa toxin and 10 μM CFTR_inh_-172 without and with 30 μM cinacalcet pretreatment for 20 minutes. (**D**) Summary of experiments as in **C** showing Δ I_SC_ following STa toxin (left) and CFTR_inh_-172 (right). (**E**) I_SC_ traces showing responses to 10 nM vasoactive intestinal peptide (VIP) and 10 μM CFTR_inh_-172 without and with 30 μM cinacalcet pretreatment for 20 minutes. (**F**) Summary of experiments as in **E** showing Δ I_SC_ following VIP and CFTR_inh_-172. *n* = 4–5 experiments per group. Mean ± SEM, Student’s *t* test, *P < 0.05, **P < 0.01, ***P < 0.001.

The cinacalcet effect on Cl^–^ secretion in T84 cells was also studied in response to 2 biologically relevant enterotoxins that cause secretory diarrhea: cholera toxin and heat-stable *E*. *coli* enterotoxin (STa toxin). Cholera toxin produced a slow but large increase in I_SC_, whereas the STa toxin effect was faster but of smaller magnitude. Cinacalcet pretreatment significantly inhibited the maximal I_SC_ increase induced by cholera toxin (Figure 5, A and B) and STa toxin (Figure 5, C and D) as well as the CFTR_inh_-172 responses.

Vasoactive intestinal peptide–secreting (VIP-secreting) neuroendocrine tumors (VIPomas) are rare causes of severe secretory diarrhea also known as “pancreatic cholera.” In this condition high circulating VIP levels stimulate intestinal fluid secretion by elevating cyclic nucleotides in enterocytes (21). Treatment of T84 cells with VIP caused an immediate and large increase in I_SC_. Cinacalcet pretreatment significantly reduced VIP-induced I_SC_ and the CFTR_inh_-172 effect (Figure 5, E and F).

### Cinacalcet activates PLC and reduces cAMP levels in T84 cells through PDEs.

CaSR can be coupled to various G proteins and signaling pathways in different tissues (6). In rat colonocytes, CaSR was shown to be functionally coupled to Gα_q_ and PLC, causing IP_3_ generation and Ca^2+^ release from intracellular stores with consequent PDE activation and cAMP breakdown (7, 8). This proposed signaling mechanism was tested in human T84 cells. Cinacalcet produced a rapid and marked increase in intracellular Ca^2+^ as measured by Fluo-4 fluorescence, which was abolished by pretreatment with the PLC inhibitor U73122 (Supplemental Figure 2, A and B).

Because cAMP is the major activator of CFTR, increased PDE activity via CaSR activation might explain the inhibitory effect of cinacalcet on CFTR-mediated Cl^–^ secretion as well as on cAMP-activated basolateral K^+^ channels. Consistent with this mechanism, cinacalcet pretreatment significantly reduced forskolin-induced elevations in cAMP (Supplemental Figure 2C). The cinacalcet effect was completely reversed by the nonselective PDE inhibitor IBMX. Together, these results suggest PLC-mediated intracellular Ca^2+^ elevation and PDE activation as the antisecretory mechanism of cinacalcet in T84 cells.

### Cinacalcet inhibits fluid accumulation in closed intestinal loop models of cholera and traveler’s diarrhea in mice.

**Figure 6:**
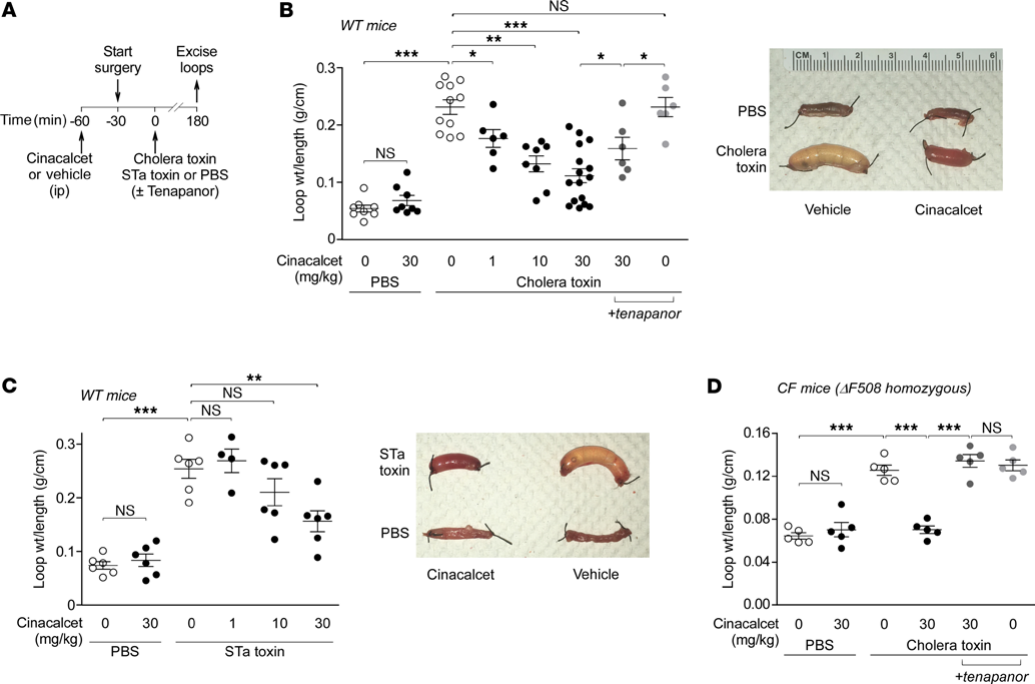
Cinacalcet prevents cholera toxin–induced and STa toxin–induced fluid accumulation in intestinal closed loops in mice. (**A**) Experimental protocol for measurement of fluid accumulation in midjejunal closed loops in mice. (**B**) Weight/length ratio and representative photos of loops injected with phosphate-buffered saline (PBS) or 1 μg cholera toxin (± 10 μM tenapanor) in WT mice pretreated with cinacalcet (1, 10, or 30 mg/kg, ip) or vehicle. *n* = 6–16 loops per group. (**C**) Weight/length ratio and representative photos of loops injected with PBS or 0.1 μg STa toxin in WT mice pretreated with cinacalcet (1, 10, or 30 mg/kg, ip) or vehicle. *n* = 4–6 loops per group. (**D**) Weight/length ratio of loops injected with PBS or 1 μg cholera toxin (± 10 μM tenapanor) in cystic fibrosis (CF, ΔF508 homozygous) mice pretreated with cinacalcet (30 mg/kg, ip) or vehicle. *n* = 5 loops per group. Mean ± SEM, 1-way ANOVA with Newman-Keuls multiple comparisons test, *P < 0.05, **P < 0.01, ***P < 0.001.

Motivated by its antisecretory action in T84 cells, we tested cinacalcet in mouse models of cholera and traveler’s diarrhea in which CFTR activation is the major secretory pathway following exposure to cholera toxin or STa toxin, respectively. Closed loop studies were done in mouse midjejunum in which cinacalcet was administered intraperitoneally prior to loop creation and instillation of toxin (Figure 6A). In cholera toxin–injected loops, cinacalcet at 30 mg/kg inhibited intestinal fluid accumulation over 3 hours (assessed by loop weight/length ratio) by approximately 75%, with significant inhibition also seen at lower doses of 1 and 10 mg/kg (Figure 6B). Cinacalcet was also effective in closed loops containing STa toxin, with approximately 60% inhibition of intestinal fluid accumulation over 3 hours at 30 mg/kg (Figure 6C).

### Cinacalcet stimulates fluid absorption from mouse small intestine.

In secretory diarrheas, intestinal fluid accumulation in response to enterotoxins involves elevation in cyclic nucleotides that results in fluid secretion due to CFTR activation and reduced fluid absorption due to NHE3 inhibition (2, 3). An earlier study showed that CaSR activation can stimulate fluid absorption from rat colonic crypts through increased NHE activity (8). In order to avoid the confounding effect of absorption in the cholera model and directly demonstrate cinacalcet action on fluid secretion, jejunal closed loops were injected with the nonabsorbable NHE3 inhibitor tenapanor together with cholera toxin. Interestingly, cinacalcet was less effective in preventing fluid accumulation in the presence of tenapanor (Figure 6B). To determine the effect of cinacalcet on NHE3 in the cholera model, closed loop experiments were done in cystic fibrosis (CF, ΔF508 homozygous) mice lacking functional CFTR. In CF mice, cholera toxin induced mild fluid accumulation as seen by the modestly increased loop weight/length ratio (Figure 6D) compared with the WT mice (Figure 6B). Cinacalcet prevented fluid accumulation in jejunal closed loops in CF mice, and this effect was abolished in the presence of luminal tenapanor (Figure 6D). These results suggest that the cinacalcet effect in the cholera model is largely due to CFTR inhibition and in part due to increased NHE3 activity.

**Figure 7:**
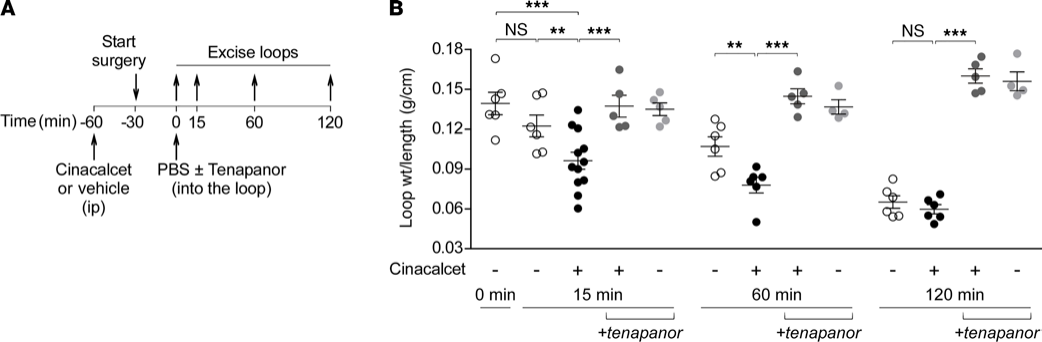
Cinacalcet stimulates fluid absorption in intestinal closed loops in mice. (**A**) Experimental protocol for measurement of fluid absorption in midjejunal closed loops in mice. (**B**) Weight/length ratio of loops injected with PBS (± 10 μM tenapanor) and removed at 0, 15, 60, or 120 minutes in mice pretreated with cinacalcet (30 mg/kg, ip) or vehicle. *n* = 4–12 loops per group. Mean ± SEM, 1-way ANOVA with Newman-Keuls multiple comparisons test, **P < 0.01, ***P < 0.001.

Experiments were also done in jejunal closed loops in the absence of secretagogues in order to further characterize the cinacalcet effect on intestinal fluid absorption (22). Closed midjejunal loops were injected with phosphate-buffered saline (PBS), and fluid absorption was determined at 15, 60, and 120 minutes (Figure 7A) by quantifying the reduction in loop weight/length ratio. In this setting fluid absorption occurred over time with complete absorption seen at 120 minutes (Figure 7B). Cinacalcet pretreatment accelerated fluid absorption from mouse jejunum as seen by lower loop weight/length ratios at 15 and 60 minutes compared with loops in vehicle-treated mice (Figure 7B). We previously showed in mouse jejunum that NHE3 is the main driver of fluid absorption in the closed loop model, in which luminal tenapanor completely blocked the reduction in loop weight/length ratio (23). Inclusion of tenapanor in the jejunal closed loops reversed the cinacalcet effect and blocked fluid absorption at all time points (Figure 7B). These data support the conclusion that cinacalcet stimulates in vivo intestinal fluid absorption through NHE3.

## Discussion

Here we showed that CaSR regulates CFTR-mediated Cl^–^ secretion in human T84 cells, and the FDA-approved CaSR activator cinacalcet prevents intestinal fluid accumulation in mouse models of cholera and traveler’s diarrhea. In humans, cinacalcet is administered at doses up to 180 mg per day (approximately 2.5 mg/kg for a 70 kg adult) for secondary hyperparathyroidism and at higher doses up to 360 mg per day for parathyroid carcinoma (24). Cinacalcet treatment in mice had a marked antisecretory action in closed intestinal loop models of cholera and traveler’s diarrhea at 30 mg/kg, a dose previously used in rodent studies (25) and equivalent to 2.5 mg/kg in humans by consideration of the body surface area conversion factor (26). Cinacalcet is thus predicted to have antidiarrheal effect in human secretory diarrheas at usual therapeutic doses, supporting its potential repurposing for major secretory diarrheas.

Consistent with earlier studies in rat colonocytes (7, 8), we found here in human T84 cells that CaSR activation by cinacalcet activated PLC to elevate intracellular Ca^2+^, resulting in PDE activation and cAMP degradation. Because cAMP is the major activator of CFTR, this mechanism is likely in large part responsible for the cinacalcet-mediated suppression of Cl^–^ secretion in T84 cells. In mice, cinacalcet also stimulated intestinal fluid absorption in an NHE3-dependent manner. Because cAMP inhibits NHE3 activity (3), the stimulatory effect of cinacalcet on intestinal fluid absorption is likely explained by reduced cAMP levels as well. In secretory diarrheas, elevation of intracellular cyclic nucleotides is the key mechanism of diarrhea. Cinacalcet might thus exert its antidiarrheal effect through multiple mechanisms, including inhibition of fluid secretion and stimulation of fluid absorption.

CaSR is also expressed in enteric neurons. Earlier studies suggested that CaSR modulates the regulatory effect of the enteric nervous system (ENS) on intestinal fluid secretion in secretory diarrhea (27). Although cinacalcet had marked antisecretory effect in T84 cells where enteric nerves are absent, its antisecretory actions in mouse intestine in vivo might also involve modulation of ENS activity. In addition to cholera or traveler’s diarrhea, cinacalcet may be effective in other forms of secretory diarrheas, such as VIPoma, as suggested by its efficacy in reducing VIP-induced secretory response in T84 cells and by its mechanism involving reducing cAMP levels in enterocytes. Although somatostatin analogs such as octreotide and/or surgical resection are the standard of care for VIPoma (28), cinacalcet may offer an alternative option for patients who do not tolerate or respond to these treatments. Other potential indications for cinacalcet include medullary thyroid cancer–associated diarrhea (29), bile acid diarrhea (30), and tyrosine kinase inhibitor diarrhea (19), in which increased CFTR-mediated Cl^–^ secretion is a major cause of diarrhea. Cinacalcet may also be effective in some congenital diarrheas such as gain-of-function mutations in *GUCY2C*, which is associated with excessive cyclic nucleotide-mediated CFTR activation in enterocytes (31), as well as microvillus inclusion disease, which may involve impaired intestinal absorption and unbalanced CFTR-mediated Cl^–^ secretion (32).

In addition to gut, CaSR is expressed in various organs, including parathyroid gland, kidney, and bone (6). Cinacalcet has good oral bioavailability and is thus expected to produce systemic effects, including hypocalcemia due to PTH suppression. Cinacalcet-induced hypocalcemia is generally asymptomatic, transient, and easily managed by dose adjustment or oral calcium supplementation (33). For short-term use as in cholera, hypocalcemia may be of lesser concern and can be prevented, if needed, by calcium supplementation. However, cinacalcet use in chronic diarrheas might be problematic due to sustained PTH suppression, which can impair bone mineralization even if hypocalcemia is prevented with calcium supplementation. Interestingly, we found here that cinacalcet exerts its effect with apical membrane administration in T84 cells. Therefore, nonabsorbable CaSR activators acting from the gut lumen can potentially have antidiarrheal efficacy without systemic action.

CaSR is expressed in both apical and basolateral membranes of human and rodent intestinal epithelium (7, 18). We previously showed efficacy of intraperitoneally administered modulators of apical membrane CFTR in mouse models of intestinal fluid transport (closed loops) and constipation (22, 34). Thus, the efficacy of intraperitoneally administered cinacalcet in mice here may result from its action on both basolateral and apical CaSR. To our knowledge, there are no reports of the apical versus basolateral membrane distribution of CaSR in T84 cells. The lack of cinacalcet efficacy when added to the basal surface of T84 cells may be due to low basolateral CaSR expression or perhaps limited physical access of cinacalcet through the porous filter because of its high hydrophobicity. Since cinacalcet is thought to bind to the transmembrane domain of CaSR (9), cellular uptake may be required for its action, in which case the apical versus basolateral location of CaSR may not be an important determinant in its action.

In conclusion, we demonstrated that the FDA-approved drug cinacalcet suppressed CFTR-mediated Cl^–^ secretion in human colonic T84 cells and prevented diarrhea in mouse models of cholera and traveler’s diarrhea. Cinacalcet or other calcimimetics can be lifesaving and logistically feasible treatments for cholera and offer an alternative treatment option for many other types of secretory diarrheas.

## Methods

### Chemicals.

All chemicals were purchased from MilliporeSigma unless otherwise specified.

### Cell culture.

T84 cells (ATCC CCL-248, human colon carcinoma cells) were cultured in a 1:1 mixture of DMEM/Ham’s F12 medium supplemented with 10% fetal bovine serum, 2 mM l-glutamine, 100 U/mL penicillin, and 100 μg/mL streptomycin. T84 cells were grown on inserts (12 mm diameter, 0.4 μm polyester membrane; Corning Life Sciences) at 37°C in 5% CO_2_/95% air and used for I_SC_ experiments 7 days after plating. FRT cells (obtained from UCSF Cystic Fibrosis Drug Discovery Core Center) stably expressing human WT CFTR (FRT-CFTR cells) were cultured as described (34) on inserts and used for I_SC_ experiments 5 days after plating. Well-differentiated HBE cells (obtained from UCSF Cystic Fibrosis Drug Discovery Core Center) were cultured at an air-liquid interface on inserts as described (22). HBE cells were used for I_SC_ experiments 21 days after plating, when they typically form a tight epithelium (*R*_TE_ > 1000 Ω cm^2^).

### I_SC_ measurements.

Cells were mounted in Ussing chambers with each hemichamber containing bicarbonate-buffered Ringer’s solution (pH 7.4, in mM: 120 NaCl, 5 KCl, 1 MgCl_2_, 1 CaCl_2_, 10 d-glucose, 5 HEPES, and 25 NaHCO_3_). Unless otherwise specified, secretagogues and ion transport modulators were added to both apical and basolateral bathing solutions. Some experiments were done using Ca^2+^- and Mg^2+^-free Ringer’s solution with and without EDTA to chelate Ca^2+^ and Mg^2+^. The solutions were aerated with 95% O_2_/5% CO_2_ and maintained at 37°C during experiments. I_SC_ was measured using an EVC4000 multichannel voltage clamp (World Precision Instruments) via Ag/AgCl electrodes and 3 M KCl agar bridges.

In some experiments to measure apical Cl^–^ conductance directly, the basolateral membrane was permeabilized with 250 μg/mL amphotericin B, and a basolateral-to-apical Cl^–^ gradient was applied, as described (22, 34), in which Ringer’s was the basolateral bathing solution (120 mM NaCl) with the apical solution containing 60 mM NaCl and 60 mM Na gluconate. To measure basolateral membrane K^+^ conductance, an apical-to-basolateral potassium gradient was applied, and the apical membrane was permeabilized with 20 μM amphotericin B (20). The apical solution (pH 7.4) contained in mM: 142.5 K-gluconate, 1 CaCl_2_, 1 MgCl_2_, 0.43 KH_2_PO_4_, 0.35 Na_2_HPO_4_, 10 HEPES, and 10 d-glucose. In the basolateral solution (pH 7.4) 142.5 mM K-gluconate was replaced by 5.5 mM K-gluconate and 137 mM N-methylglucamine.

### Intracellular Ca^2+^ and cAMP measurement.

T84 cells were plated in 96-well, black-walled microplates (Corning Life Sciences) and used at 72 hours after plating. Confluent cells were loaded with calcium indicator Fluo-4 NW (Invitrogen, Thermo Fisher Scientific) per manufacturer’s instructions. Fluo-4 fluorescence was measured in each well continuously with a Tecan Infinite M1000 plate reader (Tecan Group) at excitation/emission wavelengths of 495 nm/516 nm after manual addition of 30 μM cinacalcet (or 1% DMSO vehicle control). In some studies cells were pretreated with PLC inhibitor U73122 (10 μM) for 5 minutes prior to addition of cinacalcet. For cAMP assay, T84 cells were grown in clear 24-well plates and pretreated for 20 minutes with 30 μM cinacalcet with or without 500 μM IBMX or vehicle control (0.2% DMSO). Then cells were treated with 10 μM forskolin (for 5 minutes) and lysed by repeated freeze/thaw and centrifuged to remove cell debris. The supernatant was assayed for cAMP using the cAMP Parameter immunoassay kit according to the manufacturer’s instructions (R&D Systems, Bio-Techne).

### Intestinal closed loop model of cholera and traveler’s diarrhea.

Mice (male and female CD1, 8–12 weeks old, developed and bred in-house) were fasted overnight with access to 5% dextrose in water but no solid food before experiments. In some experiments, mice were treated with specified amounts of intraperitoneal cinacalcet hydrochloride (1, 10, and 30 mg/kg), or vehicle (saline containing 5% DMSO and 10% Kolliphor HS), 30 minutes prior to the start of abdominal surgery. Mice were anesthetized with isoflurane, and body temperature was maintained during surgery at 36°C–38°C using a heating pad. After a small abdominal incision to expose the small intestine, midjejunal loops (2–3 cm in length) were isolated by sutures as described (22, 34). Loops were injected with 100 μL PBS (pH 7.4, in mM: 137 NaCl, 2.7 KCl, 8 Na_2_HPO_4_, 1.8 KH_2_PO_4_, 1 CaCl_2_, 0.5 MgCl_2_) containing 1 μg cholera toxin or 0.1 μg heat-stable enterotoxin of *E*. *coli* (STa toxin, Bachem Americas Inc.) or PBS alone. In some experiments 10 μM tenapanor was injected into the loops together with cholera toxin or PBS to block NHE3-mediated fluid absorption (23). After loop injections the abdominal incision was closed with sutures, and mice were allowed to recover from anesthesia. Intestinal loops were removed at 3 hours, and loop length and weight were measured to quantify fluid secretion.

To quantify intestinal absorption from closed loops, midjejunal loops were isolated in mice treated with cinacalcet (30 mg/kg) or vehicle as described above. Loops were injected with 100 μL PBS and excised immediately (0 minutes), or 15, 60, or 120 minutes after injection, and loop length and weight were measured to quantify fluid absorption (23). In some experiments 10 μM tenapanor (in 100 μL PBS) was injected into the loops to block NHE3.

### Statistics.

Experiments with 2 groups were analyzed using 2-tailed Student’s *t* test; for 3 or more groups, analysis was done with 1-way ANOVA and post hoc Newman-Keuls multiple comparisons test. *P* < 0.05 was considered statistically significant.

### Study approval.

The experimental protocols were approved by the UCSF Institutional Animal Care and Use Committee. Animals were bred in UCSF Laboratory Animal Resource Center. Animal experiments were done in adherence to the NIH *Guide for the Care and Use of Laboratory Animals* (National Academies Press, 2011).

## Author contributions

OC conceived the study and designed the experiments. AAO, PDC, and OC conducted the experiments. AAR prepared and maintained the cells. AAO, PDC, and OC analyzed the data. OC wrote the paper. ASV revised the paper. All authors read the paper and approved the submitted form.

## Supplementary Material

Supplemental data

## Version 1 01/05/2021

In-Press Preview

## Version 2 02/22/2021

Electronic publication
