# Supplementary material for: Repurposing calcium-sensing receptor agonist cinacalcet for treatment of CFTR-mediated secretory diarrheas
Source: JCI Insight. 2021 Feb 22;6(4):e146823. doi: 10.1172/jci.insight.146823 (PMC7934922; doi:10.1172/jci.insight.146823)
Supplement: Supplemental data [file jciinsight-6-146823-s079.pdf]

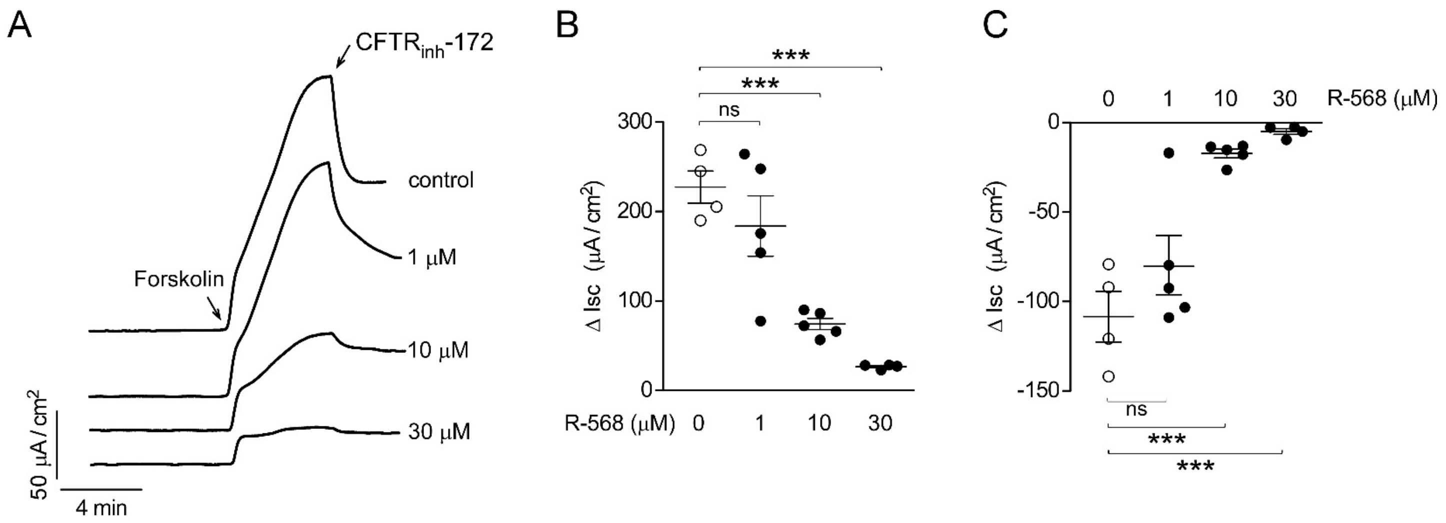

Figure S1

Figure S1. R-568 inhibits forskolin-induced Cl^- secretion in T84 cells. A. Short-circuit current (I_{sc}) traces showing maximal forskolin (10 μM) response and $CFTR_{inh}$-172 (10 μM) inhibition following 20 min pre-treatment with indicated concentrations of R-568. **B.** Δ I_{sc} induced by maximal (10 μM) forskolin at different R-568 concentrations. **C.** Δ I_{sc} following $CFTR_{inh}$-172 (10 μM) treatment at different R-568 concentrations. n=4-5 experiments per group. Mean ± S.E.M., one-way analysis of variance with Newman-Keuls multiple comparisons test, ***p<0.001, ns: not significant.

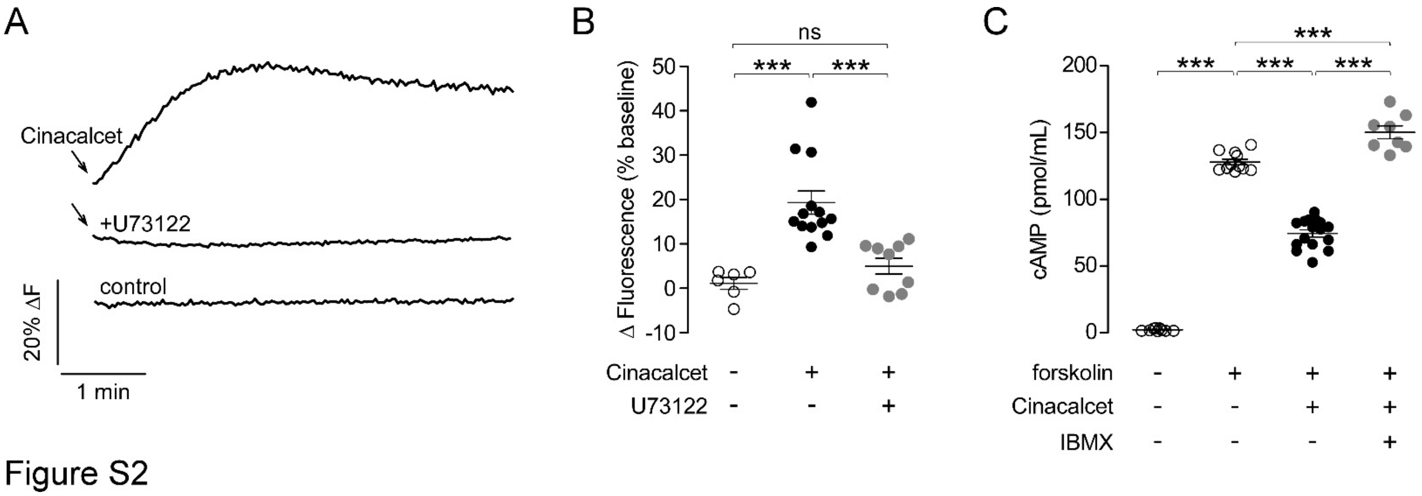

Figure S2. Cinacalcet increases intracellular Ca^{+2} via phospholipase C and inhibits forskolin-induced cAMP elevation in T84 cells. A. Intracellular Ca^{+2} traces measured by Fluo-4 NW fluorescence in T84 cells with cinacalcet (30 μM, top two traces) and vehicle control (1% DMSO, bottom) treatment at indicated times. In some experiments, T84 cells were pretreated with PLC inhibitor U73122 (10 μM) for 5 min before cinacalcet treatment (middle trace). **B.** Summary of data in A presented as maximum changes in Fluo-4 NW fluorescence as percentage of baseline reading. n=6-13 experiments per group. **C.** cAMP concentration in T84 cell lysates after maximal forskolin (10 μM) treatment with and without 30 μM cinacalcet (± 500 μM IBMX, phosphodiesterase inhibitor) pretreatment. n=8-16 experiments per group. Mean ± S.E.M., one-way analysis of variance with Newman-Keuls multiple comparisons test, ***$p<0.001$, ns: not significant.
